# Clinical Examination of the Urine and Urinary Diagnosis

**Published:** 1904-02

**Authors:** 


Clinical Examination of the Urine and Urinary Diagnosis. A Clinical Guide for the use of Practitioners and Students of Medicine and Surgery. By J. Bergen Ogden, M. D., formerly Instructor in Chemistry, Harvard University Medical School, Boston. Second revised edition. Octavo, 418 pages, illustrated, including 11 plates, 9 of them in colors. Philadelphia, New York, London: W. B. Saunders & Company. 1903. (Cloth, $3.00 net.)

The design of this work, as stated by its author, is to present in as concise a manner as possible the chemistry of the urine and its relation to physiologic processes; the most approved working methods, qualitative and quantitative; the diagnosis of diseases and disturbances of the kidneys and urinary passages.

The chief value of the book lies in its application of the information furnished by chemic and microscopic examination of urine, to urinary diagnosis. Chemic and microscopic methods take up part 1. In part 2, special attention is paid to diagnosis, the character of the urine, and the differential diagnosis of diseases of the kidney and urinary tract, local and general, surgical and medical; the clinical symptoms of each disease and the peculiarities of the urine in general diseases. In its present form, this being the second edition, brought up to date in method, it is beyond question one of the most useful and satisfying books on the subject of urinary analysis, and it cannot be too highly recommended. N. W. W.

